# Cryptocurrency trading and its relationship with other addictions among healthcare professionals in Türkiye

**DOI:** 10.7717/peerj.19314

**Published:** 2025-04-28

**Authors:** Ece Mumcu, Osman Hasan Tahsin Kılıç, Aysel Başer

**Affiliations:** 1 Department of Addiction Toxicology, Institute of Drug Abuse, Toxicology and Pharmaceutical Science, Ege University, Izmir, Turkey; 2 Department of Psychiatry, Faculty of Medicine, Izmir Democracy University, Izmir, Turkey; 3 Department of Medical Education, Faculty of Medicine, Izmir Democracy University, Izmir, Turkey

**Keywords:** Gambling, Substance use, Risky alcohol use, Cryptocurrency, Addiction, Healthcare professionals

## Abstract

**Introduction:**

There is a continuum between gambling and investing behaviors, with speculative investment instruments positioned in the middle. Cryptocurrencies, being significantly more volatile than traditional investment tools, have increasingly been linked to gambling disorder (GD). This study aims to examine the relationship between cryptocurrency trading behavior and GD, high-risk substance use, high-risk alcohol use, and tobacco dependence among healthcare professionals in Türkiye.

**Methods:**

A total of 192 healthcare professionals were assessed using the Problematic Cryptocurrency Trading Scale (PCTS), Gambling Disorder Screening Test (GDST), and the Addiction Profile Index Risk Screening Form (APIRS) (Alcohol and Drug Scales). Categorical data comparisons between two independent groups were conducted using Chi-square or Fisher’s Exact tests. Spearman correlation coefficients were used to examine relationships between PCTS scores and APIRS/GDST scores. Additionally, linear regression models assessed the predictive relationships between PCTS scores and APIRS/GDST scores.

**Results:**

Among the participants, 25.5% reported engaging in cryptocurrency trading, 41.7% had tobacco dependence, 15.1% reported high-risk alcohol use, 5.7% had high-risk substance use, and 8.9% met the criteria for GD. Cryptocurrency traders demonstrated higher rates of substance use (*p* = 0.033), tobacco dependence (*p* < 0.001), and GD (*p* = 0.043). Additionally, the severity of problematic cryptocurrency trading behavior was positively correlated with the severity of substance use (*r* = 0.172, *p* = 0.017) and GD (*r* = 0.455, *p* < 0.001).

**Conclusion:**

The findings indicate a significant relationship between cryptocurrency trading behavior and addiction. Further research with clinical interviews and larger sample sizes is required to validate these findings. The high rates of alcohol, substance, tobacco, and gambling addictions observed among healthcare professionals underscore the need for targeted preventive measures and interventions in this population.

## Introduction

Gambling is defined as an activity in which individuals risk valuables in pursuit of more valuable gains (Wildman, 1998). While stock trading and speculative investments are generally excluded from gambling research due to their skill-based nature and ties to financial enterprise fundamentals (Arthur, Williams & Delfabbro, 2016), emerging evidence suggests parallels between gamblers and speculative day traders. These similarities, particularly in demographic features and decision-making strategies, have prompted new research into speculative investment behaviors (Dixon et al., 2018; Arthur, Delfabbro & Williams, 2016).

There appears to be a spectrum between gambling and investing behaviors, with speculative financial instruments like cryptocurrencies occupying a middle ground (Arthur, Williams & Delfabbro, 2016). Cryptocurrencies, characterized by extreme volatility, have been increasingly linked to gambling behaviors (Delfabbro et al., 2021). Recent studies suggest that some cryptocurrency traders (CTs) exhibit pathological patterns of investment, potentially leading to cryptocurrency addiction—a behavior considered a subtype of gambling disorder (GD) (Guglielmo, Ioime & Janiri, 2016). Although cryptocurrencies are only legal as an investment tool in our country (not legal for use in shopping), they have been widely accepted. According to a report, 20% of Turks own cryptocurrencies, the highest per capita ownership rate in the world (Partz, 2019).

Gambling disorder is defined as a persistent and recurrent pattern of gambling behavior that disrupts interpersonal, familial, and occupational functioning (American Psychiatric Association, 2013). This disorder causes not only profound psychological and economic harm to individuals but also represents a significant public health concern with wide-ranging societal implications (Abbott, 2020). Beyond financial losses, GD is associated with severe consequences such as reduced emotional well-being, compromised physical and mental health, disrupted social and cultural dynamics, diminished workplace productivity, and increased involvement in criminal activity (Langham et al., 2016; Manu et al., 2024; Oksanen et al., 2022a). Individuals with GD are also more likely to experience suicidal thoughts and attempts compared to those without gambling problems (Armoon et al., 2023).

Notably, GD frequently coexists with substance use disorders (SUDs) and psychiatric conditions, which share similar pathophysiological mechanisms, such as neurotransmitter system abnormalities (Awaworyi Churchill & Farrell, 2018; Rash, Weinstock & Van Patten, 2016; Belfiore et al., 2024). Nearly all pathological gamblers have at least one comorbid psychiatric disorder, with SUDs being particularly prevalent (70% nicotine and 50% alcohol or drug use) (Foley, Karlsen & Putnins, 2019).

Healthcare workers represent a vulnerable population for addictions due to intense work-related stress, irregular schedules, and significant emotional pressures (Oreskovich et al., 2012; Amirouche et al., 2023). These challenges, coupled with easy access to misused prescription drugs such as opioids and sedatives, create conditions conducive to SUDs (Erkan, Erkan & Bayram, 2022; Grinspoon, 2022). Furthermore, the cultural emphasis within the medical profession on resilience and independence often deters healthcare professionals from seeking help due to stigma or fear of reputational harm (Vayr et al., 2019; Murri et al., 2021; Grinspoon, 2022).

The combination of a high-stress work environment, burnout, and psychological strain among healthcare workers may increase tendencies toward maladaptive coping mechanisms, including behaviors linked to SUDs and GD (Kenna & Lewis, 2008; Oreskovich et al., 2015; Amirouche et al., 2023). These vulnerabilities may also predispose healthcare professionals to potentially addictive behaviors like problematic cryptocurrency trading. Such behavior, considered a potential subtype of GD, could serve as a maladaptive coping mechanism to counterbalance the demands and pressures of their profession (Foley, Karlsen & Putnins, 2019).

Although the overlap between GD and SUDs—especially with nicotine, alcohol, and drug use—is well-documented, the relationship between cryptocurrency trading and SUDs remains unexplored (Rassool, 2011; Ali et al., 2023). This study aims to investigate the intricate nexus between GD, substance abuse, alcohol use, and the emerging phenomenon of cryptocurrency trading among healthcare professionals.

## Materials and Methods

### Participants and procedure

The sample of our research consists of 192 healthcare professionals from İzmir Democracy University Buca Seyfi Demirsoy Training and Research Hospital, Türkiye. Participation in the research was voluntary, and ethics committee approval was obtained from the Ethics Committee of İzmir Democracy University Buca Seyfi Demirsoy Training and Research Hospital. This study was conducted between December 2021 and May 2022. Those with a Gambling Disorder Screening Test (GDST) score of 4 or above were classified as having GD. Participants with an Addiction Profile Index Risk Screening (APIRS) alcohol score of 3 or above were categorized as high-risk alcohol users, and those with an APIRS drug score of 4 or above were classified as high-risk substance users.

### Screening and selection process

The target population of the study consists of 1,193 employees working at a training and research hospital in İzmir. For the study, an initial sample of 362 healthcare workers was selected as potential participants using a purposive sampling method based on their profession, age, and gender. A preliminary screening was conducted to assess whether these individuals met the study criteria, and eligible participants were subsequently invited to take part in the study. The inclusion criteria for the study were being a hospital employee and voluntarily consenting to participate. Individuals with chronic systemic diseases were excluded from the study, as these conditions could influence patterns of tobacco, alcohol, and substance use.

However, 170 individuals were excluded for various reasons during the screening process. Among them, 36 had work schedules that conflicted with the study timings, 24 lacked interest in the research topic, 62 were unable to complete the scale forms during screening, and 48 were excluded due to personal health issues. Participants were required not to have been diagnosed with any chronic physical illness at the beginning of the study. This criterion was important to ensure that the addiction behaviors examined in the research were not influenced by chronic illness conditions. Of the 300 individuals contacted, 192 agreed to participate in the study. All participants who volunteered for the study were over 18 years of age and provided both written and verbal informed consent.

### Adequacy of sample size

The sample of 192 healthcare professionals represents 1.6% of the hospital staff. This sample size is adequate for the analyses conducted in our study. The sample size calculation accounted for the statistical power necessary to detect relationships between the study’s primary variables. Furthermore, our sample encompasses the broad demographic and professional diversity of the hospital staff, allowing the results to be reasonably generalized to the overall population of hospital employees.

### Data collection tools

The research employed four primary tools: the Sociodemographic Data Form, the Problematic Cryptocurrency Trading Scale (PCTS), the Gambling Disorder Screening Test (GDST), and the Addiction Profile Index Risk Screening (APIRS). All scales used in the study have been validated and shown to be reliable in Turkish samples, ensuring cultural and linguistic appropriateness (Evren et al., 2020; Menteş, Yolbaş & Bulut, 2021; Ögel, Koç & Görücü, 2017).

#### Sociodemographic data form

Developed by the researchers, this form includes questions on participants’ age, gender, education level, income, marital status, parenthood, occupation, smoking status, cryptocurrency trading behavior, gambling behavior, and psychiatric history.

#### Problematic Cryptocurrency Trading Scale (PCTS)

The Problematic Cryptocurrency Trading Scale (PCTS), developed by Menteş, Yolbaş & Bulut (2021), measures problematic cryptocurrency trading behaviors. It comprises 15 items scored on a 5-point Likert scale (1 = Never, 5 = Always) and includes two sub-dimensions: “Withdrawal and Tolerance” (11 items) and “Money-Finding Behavior and Denial” (four items). While a cut-off score was not established in the original study, Cronbach’s alpha value for the total score was reported as 0.913, indicating high reliability. In this study, Cronbach’s alpha values were 0.943 for the total score and 0.952 and 0.562 for the two sub-dimensions, respectively.

#### Gambling Disorder Screening Test (GDST)

The GDST, developed by Evren et al. (2020), is based on the nine criteria for Gambling Disorder as defined in the DSM-5. The scale consists of 11 items, with responses coded as “Never” (0), “Yes, at some point in my life” (1), “Yes, last year” (2), and “Yes, last 3 months” (3). Items 8, 9, and 10 are evaluated as a single item, and scores range from 0 to 9. A diagnosis is made when four or more criteria are met. The GDST’s original Cronbach’s alpha value was 0.93, indicating high internal reliability, which was confirmed in this study with a Cronbach’s alpha value of 0.923 (Evren et al., 2020).

#### Addiction Profile Index Risk Screening (APIRS)

The APIRS, developed by Ögel, Koç & Görücü (2017), assesses the risk levels for alcohol and substance use. The tool includes two sub-scales: a six-item section for alcohol use and a seven-item section for substance use. Scores of 3 or more on the alcohol scale and 4 or more on the drug scale indicate a high risk. The original reliability scores (Cronbach’s alpha) were 0.7 for alcohol and 0.88 for drugs. In this study, the reliability scores were 0.755 for alcohol and 0.875 for drugs, demonstrating acceptable internal consistency.

### Ethics approval and consent to participate

Ethical approval for the study was obtained from the Non-Interventional Clinical Research Ethics Committee of İzmir Democracy University Faculty of Medicine, Buca Seyfi Demirsoy Training and Research Hospital (Decision No: 2021/11-67, Date: 24/11/2021).

### Statistical analysis

In this study, the demographic characteristics of participants, such as gender, marital status, educational level, parenthood status, and age, were analyzed using descriptive statistics. This included calculating means, standard deviations, frequencies, and percentage distributions to provide a clear understanding of the sample’s profile. Since the numerical data did not fit a normal distribution, we employed non-parametric tests. In the comparison of categorical data between two independent groups, the appropriate Chi-square or Fisher’s exact test was utilized. Furthermore, the study used the Spearman correlation coefficients to assess the relationship between scores from the Problematic Cryptocurrency Trading Scale (PCTS) and those from the Addiction Profile Index Risk Screening (APIRS) for alcohol and drugs, as well as the Gaming Disorder Screening Test (GDST). A linear regression model was applied to analyze the relationships between the PCTS scores and the APIRS and GDST scores. Separate regression plots were created for each addiction variable to visualize the linear relationship between the variables. In the regression model, the APIRS-drug total score and GDST total score were included as dependent variables, while the PCTS total score was included as the independent variable. The coefficient r indicates the magnitude of the correlation, and the sign in front of it (+ or −) indicates the direction of the correlation. Significance levels for all tests were set at *p* < 0.05, indicating a 95% confidence interval for the statistical significance of the findings. Analyses were conducted using IBM SPSS v25.0 (SPSS Inc., Chicago, IL, USA).

## Results

Of the 192 participants, 120 (62.5%) were female, 104 (59.4%) were married, 102 (53.1%) were university graduates, and 105 (54.7%) had children, with a mean age of 40.75 ± 15.33. The study included a diverse group of healthcare professionals, with 53 doctors (28%), 87 nurses (45%), and 52 participants (27%) from other professions such as physiotherapists, psychologists, and laboratory technicians, highlighting broad professional representation.

According to the APIRS-alcohol and APIRS-drug scores, 29 participants (15.1%) exhibited high-risk alcohol use, and 11 (5.7%) exhibited high-risk substance use. Based on GDST scores, gaming disorder (GD) was identified in 17 participants (8.9%). Additionally, 80 participants (41.7%) reported smoking daily, and 49 (25.5%) reported trading cryptocurrencies.

Cryptocurrency trading behavior was significantly higher among individuals with GD (*p* = 0.043), those who engage in sports prediction games (*p* = 0.001), legal lotteries (*p* = 0.001), casino games (*p* = 0.033), stock investments (*p* < 0.001), males (*p* = 0.003), those with children (*p* = 0.017), smokers (*p* < 0.001), and substance users (*p* = 0.033). No significant difference was observed between cryptocurrency traders and non-traders regarding high-risk alcohol use (*p* > 0.05) (Table 1). Table 1 presents a comparison of healthcare workers who trade cryptocurrency and those who do not, based on sociodemographic characteristics and other addiction behaviors.

**Table 1:** Comparison of cryptocurrency traders and non-traders.

*n* = 192 (%100)	NCT *n* = 143 (%74)	CT *n* = 49 (%25)	*p*
**Gender**			
Female 120 (%62.5)	98 (%68.5)	22 (%44.9)	**0.003** ^a^
Male 72 (%37.5)	45 (%31.5)	27 (%55.1)	
**Occupation**			
Physician 53 (%27.6)	44 (%30.8)	9 (%18.4)	
Nurse 87 (%45.3)	59 (%41.3)	28 (%57.1)	0.121^a^
Other staff 52 (%27.1)	40 (%28)	12 (%24.5)	
**Having child**			
Yes 105 (%54.7)	71 (%49.7)	34 (%69.4)	**0.017** ^a^
No 87 (%45.3)	72 (%51.2)	15 (%31.6)	
**Cigarette use**			
Yes 80 (%41.7)	47 (%32.9)	33 (%67.3)	**<0.001** ^a^
No 112 (%58.3)	96 (%69.1)	16 (%32.7)	
**GD**			
Yes 17 (%8.9)	9 (%6.3)	8 (%16.3)	**0.043** ^b^
No 175 (%91.1)	134 (%93.7)	41 (%83.7)	
**Substance use**			
Yes 11 (%5.7)	5 (%3.5)	6 (%12.2)	**0.033** ^b^
No 181 (%94.3)	138 (%96.5)	43 (%87.8)	
**Risky alcohol use**			
Yes 29 (%15.1)	18 (%12.6)	11 (%22.4)	0.096^a^
No 163 (%84.9)	125 (%87.4)	52 (%77.6)	

**Notes.**

CT Cryptocurrency traders GD Gambling Disorder NCT non-cryptocurrency traders

a Chi-square.

b Fisher’s Exact Test.

Bold values indicate statistically significant results (*p* < 0.05).

The mean APIRS-alcohol, APIRS-drug, GDST and PCTS scores were 0.937 (min:0, max:8), 0.171 (min:0, max:7), 11.604 (min:11, max:32) and 18.640 (min:15, max:67), respectively. While there was a significant and low positive correlation between PCTS scores and APIRS-drug scores (*r* = 0.172, *p* = 0.017) there was a significant and moderate positive correlation between PCTS scores and GDST scores (*r* = 0.455, *p* < 0.001) (Table 2).

**Table 2:** Correlation between PCTS and APIRS-drug, APIRS-alcohol, and GDST scores.

Correlations: problematic cryptocurrency trading scale					
			***APIRS-alcohol***	***APIRS-drug***	**GDST**
Spearman’s rho	PCTS	r	0.118	0.172^*^	0.455^**^
		*p*	0.103	0.017	0.000
		N	192	192	192

**Notes.**

* Correlation is significant at the 0.05 level (two-tailed).

** Correlation is significant at the 0.01 level (two-tailed).

APIRS Addiction profile index risk screening scale GDST Gaming Disorder Screening Test Spearman Correlation coefficients

**Figure 1:**
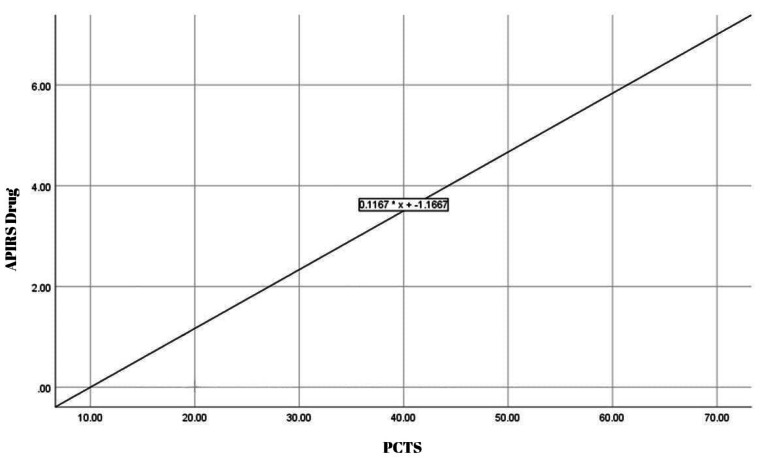
Regression plot of PCTS *vs* APIRS drug.

A positive relationship was observed between PCTS total and APIRS-drug total (*B* = 0.1167); however, the slope coefficient indicates a modest rate of increase (Fig. 1).

**Figure 2:**
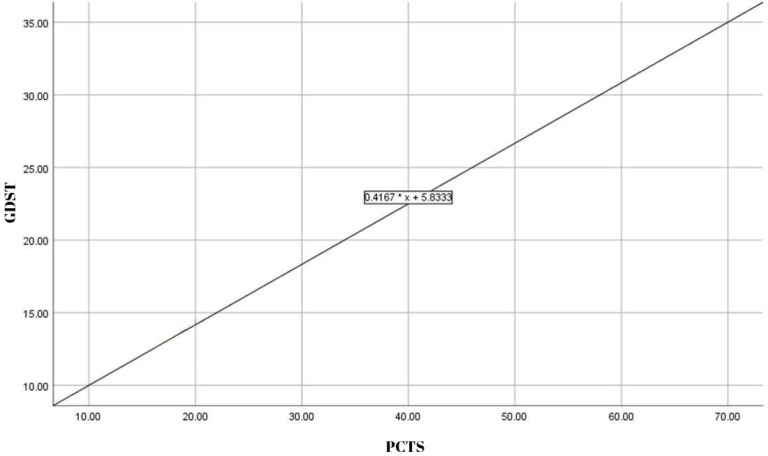
Regression plot of PCTS *vs* APIRS drug.

Similarly, a positive relationship was identified between PCTS total and GDST total (*B* = 0.4167), suggesting that higher PCTS total scores are associated with a significant increase in GDST total scores (Fig. 2). These findings suggest that problematic cryptocurrency trading may influence the risk of gambling disorder and substance use, although the strength of these relationships appears to be relatively low.

## Discussion

This is the first study to examine the relationship between problematic cryptocurrency trading behavior and high-risk alcohol use, substance use, tobacco dependence, and GD among healthcare professionals in Türkiye. Approximately 25% of the healthcare professionals engaged in cryptocurrency trading, 41.7% had tobacco dependence, 15.1% exhibited high-risk alcohol use, 5.7% reported substance use, and 8.9% met the criteria for GD. Substance use, tobacco dependence, GD, betting on sports games, playing the lottery, casino gaming, and stock market trading were more prevalent among cryptocurrency traders. However, there was no significant difference between cryptocurrency traders and non-traders in terms of high-risk alcohol use. The severity of problematic cryptocurrency trading behavior was positively correlated with the severity of substance use disorders (SUDs) and GD.

Previous studies have reported a relationship between cryptocurrency trading and gambling (Delfabbro et al., 2021; Mills & Nower, 2019; Oksanen et al., 2022). A recent study found that high-frequency CTs had higher gambling pathology and impulsivity (Oksanen et al., 2022). Another study reported that the intensity of cryptocurrency trading, determined by time, number, and volume of trades per day, was correlated with pathological gambling scores (Delfabbro et al., 2021). Consistent with previous studies, we found that cryptocurrency trading behavior was higher in people with GD and gambling activities (betting on sports games, playing the lottery, and casino games). Similar to gambling, there is a significant element of luck involved in cryptocurrency trading. There are more uncontrollable factors than with typical financial tools, and decisions are made with less planning. Because of these shared factors, there is a growing view that cryptocurrency trading behavior resembles gambling behavior (Delfabbro et al., 2021). Our results support studies suggesting that cryptocurrency trading is a form of gambling.

Although there are no studies examining tobacco and substance use aside from alcohol in cryptocurrency traders, studies investigating the relationship between gambling behavior and substance use have reported that gamblers use more anabolic steroids and cannabis, consume cocaine, and have higher lifetime smoking rates (Hall et al., 2000; Ledgerwood & Downey, 2002; Widinghoff et al., 2019). Additionally, it was reported that smokers gamble more, spend more money on gambling, desire to gamble more, and have less control over their gambling behavior (Petry & Oncken, 2002). We found that substance use and tobacco dependence were higher in cryptocurrency traders. Contradictory to our results, the only study examining alcohol use in CTs reported higher excessive alcohol use (Oksanen et al., 2022). However, we did not find any difference between traders and non-traders in terms of high-risk alcohol use. Although high-risk alcohol use was higher in CTs, our findings were not statistically significant. This may be due to the small sample size.

In studies examining the sociodemographic characteristics of CTs, it has been consistently reported that cryptocurrency trading behavior is higher in males. As we expected, we found a higher rate of CT in men and suggest that this may be related to higher impulsivity in males. Conflicting results have been reported regarding age and marital status (Mills & Nower, 2019; Oksanen et al., 2022). We did not find any association between cryptocurrency trading behavior and age or marital status. Interestingly, we found higher rates of cryptocurrency trading in those with children, unlike a previous study in Finland (Balodis & Potenza, 2020). Our difference from the Finnish study may be due to economic differences between the two countries.

The migration of healthcare professionals, especially physicians, from Türkiye has been an issue that has attracted considerable attention in the last 10 years. According to the Turkish Medical Association 2022 Working Report, the migration of physicians has increased 24 times in the last 10 years (Turkish Medical Association, 2022). Low working wages and hopelessness stand out among the reasons leading to the migration of healthcare professionals in our country (Alkan et al., 2023). We speculate that higher cryptocurrency trading in those with children could be related to financial difficulties associated with having children.

This study identified high rates of gaming disorder GD, daily tobacco use, risky alcohol consumption, substance use, and cryptocurrency ownership among healthcare workers, with one in four reportedly owning cryptocurrency. Numerous studies have demonstrated a strong correlation between alcohol consumption, working hours, and workplace conditions (Amirouche et al., 2023). Given the heightened risk of developing addictions in this population, problematic cryptocurrency use may also be more prevalent among healthcare professionals (Amirouche et al., 2023; Kunyk et al., 2016). Consistent with the literature, we believe these elevated rates are linked to long working hours, challenging working conditions, and particularly low wages among healthcare workers in Türkiye.

### Limitations

Potential self-reporting biases and the single-center nature of the study limit the generalizability of our findings. Additionally, the high stress levels commonly experienced by healthcare workers, which may increase the risk of addiction, could act as a confounding factor. Another limitation is the absence of a standardized cut-off value for the PCTS, which complicates the differentiation between problematic and non-problematic cryptocurrency trading behaviors. Future studies should address these limitations by utilizing larger, multi-center samples and incorporating diverse data collection methods to enhance the applicability and generalizability of our findings to broader populations.

## Conclusion

This study identified a significant association between cryptocurrency trading and other addiction-related disorders, including smoking, GD, and substance use, among healthcare professionals. These findings suggest the potential classification of cryptocurrency trading as a subtype of gambling disorders within the broader spectrum of addictive behaviors. The results highlight the need for future research with larger samples, diverse occupational groups, and longitudinal study designs to better understand the connections between cryptocurrency trading and other forms of addiction. Our study emphasizes the association between cryptocurrency trading and addictive behaviors, as well as the high prevalence of addiction among healthcare professionals. These findings underscore the urgent need for preventive measures in this field. Effective prevention strategies should include psychological support programs, improved working conditions, institutional support, training initiatives, physical health promotion, and robust social support systems. Achieving this requires the development of multidisciplinary strategies that foster collaboration among mental health professionals, social workers, occupational health specialists, and professional associations. These findings highlight the urgent need for targeted preventive interventions within this population to effectively address addiction risks (Bruschwein & Gettle, 2020).

##  Supplemental Information

10.7717/peerj.19314/supp-1 Supplemental Information 1 Data

10.7717/peerj.19314/supp-2 Supplemental Information 2 Code

## Additional Information and Declarations

### Data Availability

The following information was supplied regarding data availability: The data that support the findings of this study are available at Zenodo: Kılıç, Osman Hasan Tahsin (2024). Cryptocurrency Trading and Its Relationship with Other Addictions Among Healthcare Professionals: Cross Sectional Study [Data set]. Zenodo. https://doi.org/10.5281/zenodo.11535481.
